# Twenty-five-year mortality trends of four major histological subtypes of cervical cancer: a population-based study using the Osaka cancer registry data

**DOI:** 10.3389/fonc.2023.1233354

**Published:** 2023-11-27

**Authors:** Naoko Komura, Seiji Mabuchi, Tomoyuki Sasano, Shoji Kamiura, Toshitaka Morishima, Isao Miyashiro

**Affiliations:** ^1^ Department of Obstetrics and Gynecology, Kaizuka City Hospital, Osaka, Japan; ^2^ Department of Gynecology, Osaka International Cancer Institute, Osaka, Japan; ^3^ Department of Obstetrics and Gynecology, Osaka Saiseikai Nakatsu Hospital, Osaka, Japan; ^4^ Cancer Control Center, Osaka International Cancer Institute, Osaka, Japan

**Keywords:** cervical cancer, population-based study, 25 years, mortality trends, osaka cancer registry

## Abstract

**Objective:**

To assess the mortality trends of four major histological subtypes of cervical cancer diagnosed between 1994 and 2018.

**Methods:**

This population-based retrospective cohort study was conducted using the Osaka Cancer Registry data from 1994 to 2018. A total of 12,003 patients with cervical cancer, squamous cell carcinoma (SCC), adenocarcinoma (A), adenosquamous cell carcinoma (AS), or small cell neuroendocrine carcinoma (SCNEC) were identified. Patients were classified into groups according to the extent of disease (localized, regional, or distant), year of diagnosis (1994–2002, 2003–2010, or 2011–2018), and histological subtype (SCC, A/AS, or SCNEC). Then, their survival rates were assessed using univariate and multivariate analyses.

**Results:**

Overall, improved survival rates were observed according to the year of diagnosis in patients with local, regional, and distant cervical cancers. When examined according to the histological subtypes, improved survival rates according to the year of diagnosis were observed in patients with local, regional, and distant SCCs and in those with local and regional A/AS. In patients with distant A/AS, the survival rates did not improve since 2003. In patients with cervical cancer with SCNEC, the survival rates did not improve since 1994 irrespective of the extent of the disease. In the multivariate analysis, non-SCC histology was found to be an independent prognostic factor for OS.

**Conclusion:**

In contrast to SCC histology associated with improved survival between 1994 and 2018, SCNEC histology and advanced (stage IVB) A/AS remain to be the unmet medical needs for the management of cervical cancer.

## Introduction

Uterine cervical cancer is the third most common gynecologic cancer and the leading cause of death among gynecologic cancers in the United States (1). Globally, in 2020, cervical cancer was the fourth most common cancer in females with an estimated 604,000 new cancer cases and 342,000 deaths, 85% of which occurred in resource-limited regions (2).

The histological subtypes of cervical cancer have been defined according to the 2014 World Health Organization (WHO) classification (3) and the major histological subtypes include squamous cell carcinoma (SCC), adenocarcinoma with various subtypes (A), adenosquamous cell carcinoma (AS), and neuroendocrine tumor (NEC). NEC is categorized into four types: typical carcinoid, atypical carcinoid, small cell neuroendocrine carcinoma (SCNEC), and large cell neuroendocrine carcinoma, with SCNEC being the predominant subtype.

SCNEC of the uterine cervix is a rare pathologic form, constituting 0.9%–1.5% of all invasive cervical cancers and with an annual incidence rate of 0.06 per 100,000 women (4–7). Although the etiology of SCNEC of the uterine cervix, human papillomavirus infection, is the same as that of SCC, A, and AS (8), SCNEC has been associated with worse clinical outcomes than SCC or A/AS (5). In patients with early-stage cervical cancer (stages IB–IIA), the hazard ratio (HR) for death was 2.96 times higher for SCNEC compared to SCC (5). In patients with locally advanced disease (stages IIB–IVA), the HR for death was 1.70 times higher for SCNEC compared to r SCC (5). According to a previous study, the 5-year overall survival (OS) rate of SCNEC of the uterine cervix was 35.7%, which was lower than those of SCC (60.5%) and A/AS (69.7%) (4). Even in patients with stage IB disease, the 5-year OS rate for SCNEC (55.4%) was significantly lower than those for SCC (80.4%) and A/AS (85.7%) (5).

Presently, Japan is the only developed country that has experienced increasing trends of cervical cancer in both incidence and mortality in the last 10 years (9). This is partially because of the insufficient operation of cervical cancer screening programs and dissemination of human papillomavirus (HPV) vaccination (10). Although cervical cancer will remain an important health issue for the foreseeable future in Japan, trends in histological distribution, treatment, and survival has not been fully investigated. Thus, the unmet needs in the management of cervical cancer in Japan remains unclear.

With the marked advances in surgery, chemotherapy, radiotherapy, and immunotherapy, the survival of patients with cervical cancer in the early-stage, advanced-stage, and recurrent settings, especially with SCC or A/AS histology, has significantly improved in the last 25 years (11). However, because patients with SCNEC had been excluded from previous landmark studies, a novel treatment strategy specifically targeting SCNEC has not yet been established. Moreover, mainly because of the rarity of this histological subtype, the mortality trend in patients with SCNEC has not yet been fully investigated.

Based on these, we conducted a population-based study using the Osaka Cancer Registry (OCR) data between 1994 and 2018. The main objectives of the present study were to examine the impact of histological subtypes on mortality trends and to highlight the “unmet needs” in the management of cervical cancer.

## Materials and methods

### Data source

This retrospective observational study was conducted in Osaka Prefecture, Japan, using data obtained from the population-based OCR. The OCR is a full longitudinal survey that collects information on the diagnosis and treatment of all cancers occurring in Osaka Prefecture, which has been in operation since 1962 (12).

The OCR records all new cancer cases recognized by reports from medical facilities or death certificate databases. Patient data from the OCR included sex, age at cancer diagnosis, date of diagnosis, and date of death or the last vital status follow-up. Tumor-specific data included the cancer site, extent of disease, histology, and date of cancer diagnosis. The extent of the disease was classified into the following three groups: 1) localized, cancer was confined to the original organ; 2) regional, cancer had spread to regional lymph nodes and/or to immediately adjacent tissues; and 3) distant, cancer had metastasized to distant organs. The correspondence between the extent of disease and its FIGO 2008 classification was as follows: localized, stage I; regional, stages II, III, and IVA; and distant, stage IVB. The histological type was determined using the morphology code of the International Classification of Diseases for Oncology, Third Edition (ICD-O3M), and only SCC, A (endometrioid carcinoma, serous carcinoma, clear cell carcinoma, mucinous carcinoma, and adenocarcinoma not otherwise specified), AS, and SCNEC were included in the analyses. Treatment data included the type of primary treatment (i.e., surgery, chemotherapy, or radiation therapy) and the hospital at which patients were diagnosed and received treatment. However, the registry does not collect information on the patients’ socioeconomic characteristics, comorbidity history, content of follow-up treatment and care after the initial treatment, or causes of death. Follow-up of the vital status of patients with cancer is routinely performed using death certificates. Furthermore, these patients were followed up using official resident registries to verify their vital status at 3, 5, and 10 years after diagnosis (12).

### Study population

**Figure 1:**
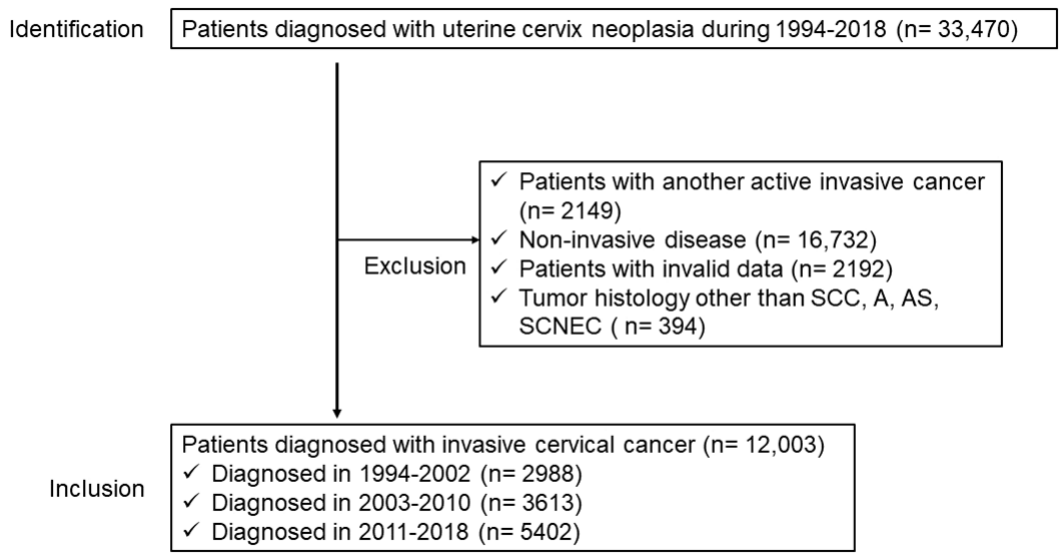
Flow diagram of the study population.

The inclusion criteria were as follows: cases of uterine cervical neoplasia (C53, malignant neoplasm of the cervical uteri) registered in the OCR from 1994 to 2018; patients with SCC, A, AS, or SCNEC histology; and patients residing in Osaka at the time of diagnosis. Age restrictions were not imposed. Patients with carcinoma in situ; histological types other than SCC, A/AS, and SCNEC; multiple cancers; or unknown extent of disease were excluded from the survival analyses. Accordingly, 12,003 women with cervical cancer were analyzed for survival (**Figure 1**). This study population did not include death-certificate-only cases.

### Statistical analysis

OS was defined as the time from the date of cervical cancer diagnosis to the date of death or the last follow-up visit. The OS rate was compared between groups using the Kaplan–Meier method, and the results were compared using the log-rank test. Continuous data were compared between groups using Student’s t-test, the Wilcoxon rank-sum test, or the median test, as applicable. Frequency counts and proportions were compared between groups using the chi-square test or Fisher’s two-tailed exact test, as applicable. Cox’s proportional hazards regression analysis was performed to identify significant independent prognostic factors for survival. All analyses were conducted using JMP version 16.0 (SAS Institute, Cary, NC, USA), and a P value of <0.05 was considered statistically significant.

## Results

### Investigations involving all patients with cervical cancer

From January 1, 1994, to December 31, 2018, 33,470 women in Osaka were diagnosed with uterine cervical neoplasm and underwent primary treatment. Study population selection is shown in **Figure 1**. In total, 12,003 patients with invasive cervical cancer displaying SCC, A, AS, and SCNEC histologies were included in the analysis (**Figure 1**). Among the excluded 2149 patients with another invasive cancer, 1517 (70.6%), 880 (17.7%), and 8 (0.4%) had SCC, A/AS, and SCNEC, respectively, which was similar to the distribution in the main study population, 9444 (78.7%), 2464 (20.5%), and 95 (0.8%) for SCC, A/AS, and SCNEC, respectively.

The clinicopathological characteristics of the patients are presented according to the year of diagnosis (**Table 1**). Of these, 5,915, 4,930, and 1,158 were diagnosed with local, regional, and distant cervical cancers, respectively. Cervical cancer was diagnosed in 1994–2002 in 2,988 patients, 2003–2010 in 3,613 patients, and 2011–2018 in 5,402 patients. The proportion of patients with A/AS histology increased from 16.7% in 1994–2002 to 21.8% in 2003–2018.

**Table 1:** Clinicopathological characteristics of all cervical cancer patients included in the current study.

		1994-2002 (Total = 2988) N (%)	2003-2010 (Total= 3613) N (%)	2011-2018 (Total= 5402) N (%)	p-value
Age	≤39	565 (18.9)	802 (22.2)	1202 (22.3)	0.0013
	40-60	1366 (45.7)	1603 (44.4)	2308 (42.7)	
	≥61	1057 (35.4)	1208 (33.4)	1892 (35.0)	
Histological subtype	SCC	2843 (83.1)	2788 (77.2)	4173 (77.3)	<0.0001
	A/AS	498 (16.7)	787 (21.8)	1179 (21.8)	
	SCNEC	7 (0.2)	38 (1.1)	50 (0.9)	
Extent of disease	Localized	1555 (52.0)	1779 (49.2)	2581 (47.8)	<0.0001
	Regional	1228 (41.1)	1497 (41.4)	2205 (40.8)	
	Distant	205 (6.9)	337 (9.3)	616 (11.4)	
Primary Treatment	Surgery	1840 (61.6)	2335 (64.6)	3070 (56.8)	<0.0001
	Radiotherapy	867 (29.0)	940 (26.0)	1595 (29.5)	
	Chemotherapy	51 (1.7)	120 (3.3)	219 (4.1)	
	BSC	213 (7.1)	180 (5.0)	466 (8.6)	
	Unknown	17 (0.6)	38 (1.1)	52 (1.0)	

SCC, squamous cell carcinoma; A, adenocarcinoma; AS, adenosquamous carcinoma; SCNEC, small cell neuroendocrine carcinoma; BSC, best supportive care.

**Figure 2:**
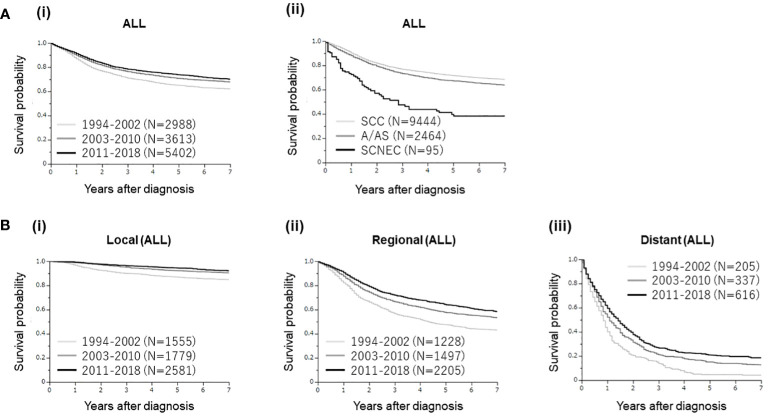
**(A)** Kaplan–Meier estimates of the overall survival of all patients with cervical cancer included in the present study according to the year of diagnosis and histological subtypes. (i) Clearly differential survival rates were observed according to the year of diagnosis (group 1 [1994–2002] vs. group 2 [2003–2010], p<0.0001; group 1 vs. group 3 [2011–2018], p<0.0001; group 2 vs. group 3, p=0.0116). (ii). Clearly differential survival rates were observed according to the histological subtypes [SCC versus A/AS; p<0.0001, SCC versus SCNEC; p<0.0001, A/AS versus SCNEC; p<0.0001]. **(B)** Kaplan–Meier estimates of the overall survival of all patients with cervical cancer included in the present study according to the disease extent and year of diagnosis. **(A)** Patients with local disease (group 1 [1994–2002 vs. group 2 [2003–2010], p<0.0001; group 1 vs. group 3 [2011–2018], p<0.0001; group 2 vs. group 3, p=0.0263). **(B)** Patients with regional disease (group 1 [1994–2002] vs. group 2 [2003–2010], p<0.0001; group 1 vs. group 3 [2011–2018], p<0.0001; group 2 vs. group 3, p=0.0004). **(C)** Patients with distant disease (group 1 [1994–2002] vs. group 2 [2003–2010], p=0.0003; group 1 vs. group 3 [2011–2018], p<0.0001; group 2 vs. group 3, p=0.0144).

In the survival analyses involving all cervical cancer patients (**Figure 2**), as shown, clearly differential survival rates were observed according to the year of diagnosis [**Figure 2A** (i)] and histological subtypes [**Figure 2A** (ii)].

When investigated according to the extent of disease, improved survival rates were observed according to the year of diagnosis in patients with local, regional, and distant cervical cancers (**Figure 2B**), indicating significant progress in cervical cancer management in Osaka, Japan.

We next conducted a multivariate analysis for OS (**Table 2**). As shown, in addition to advanced age, advanced diseases, non-local treatments and older year at diagnosis, non-SCC histology was found to be an independent prognostic factor for OS. The prognostic impact of SCNEC histology [hazard ration (HR) of 2.28, 95% confidence interval (CI) of 1.75-2.98] was higher than that of A/AS histology [hazard ratio (HR) of 1.41, 95% confidence interval (CI) of 1.30-1.52].

**Table 2:** Univariate and multivariate analysis of prognostic factors for overall survival in cervical cancer patients.

		Univariate analysis			Multivariate analysis		
		HR	95% CI	p-value	HR	95% CI	p-value
Age	≤39	1			1		
	40-60	2.26	2.02-2.54	<0.0001	1.60	1.43-1.80	<0.0001
	≥61	5.20	4.65-5.82	<0.0001	2.92	2.61-3.28	<0.0001
Histological subtype	SCC	1			1		
	A/AS	1.17	1.09-1.26	<0.0001	1.41	1.30-1.52	<0.0001
	SCNEC	2.69	1.99-3.37	<0.0001	2.28	1.75-2.98	<0.0001
Extent of disease	Localized	1			1		
	Regional	5.09	4.69-5.52	<0.0001	4.58	4.21-4.99	<0.0001
	Distant	18.53	1681-20.42	<0.0001	15.25	13.73-16.94	<0.0001
Primary Treatment	Local treatments*	1			1		
	Chemotherapy	4.39	3.88-4.95	<0.0001	1.71	1.50-1.94	<0.0001
	BSC	2.46	2.23-2.72	<0.0001	3.13	2.83-3.46	<0.0001
Year of diagnosis	2011-2018	1			1		
	2003-2010	1.13	1.05-1.22	0.0017	1.28	1.87-1.39	<0.0001
	1994-2002	1.33	1.23-1.44	<0.0001	1.78	1.64-1.93	<0.0001

HR, hazard ration; SCC, squamous cell carcinoma; A, adenocarcinoma; AS, adenosquamous carcinoma; SCNEC, small cell neuroendocrine carcinoma; BSC, best supportive care.

*Surgery or radiotherapy.

### Investigations according to the histological subtypes

Of the 12,003 patients, 9,444, 2,464, and 95 had SCC, A/AS, and SCNEC, respectively. As shown in **Supplemental Table 1**, SCNEC histology was associated with a younger age at diagnosis and distant disease. Moreover, the proportion of surgically treated patients was higher in those with A/AS histology than in those with SCC or SCNEC histology.

We then investigated the impact of the year of diagnosis on patient survival according to the histological subtype. The clinicopathological characteristics of patients with SCC, A/AS, and SCNEC according to the year of diagnosis are presented in **Supplemental Tables 2–4**.

**Figure 3:**
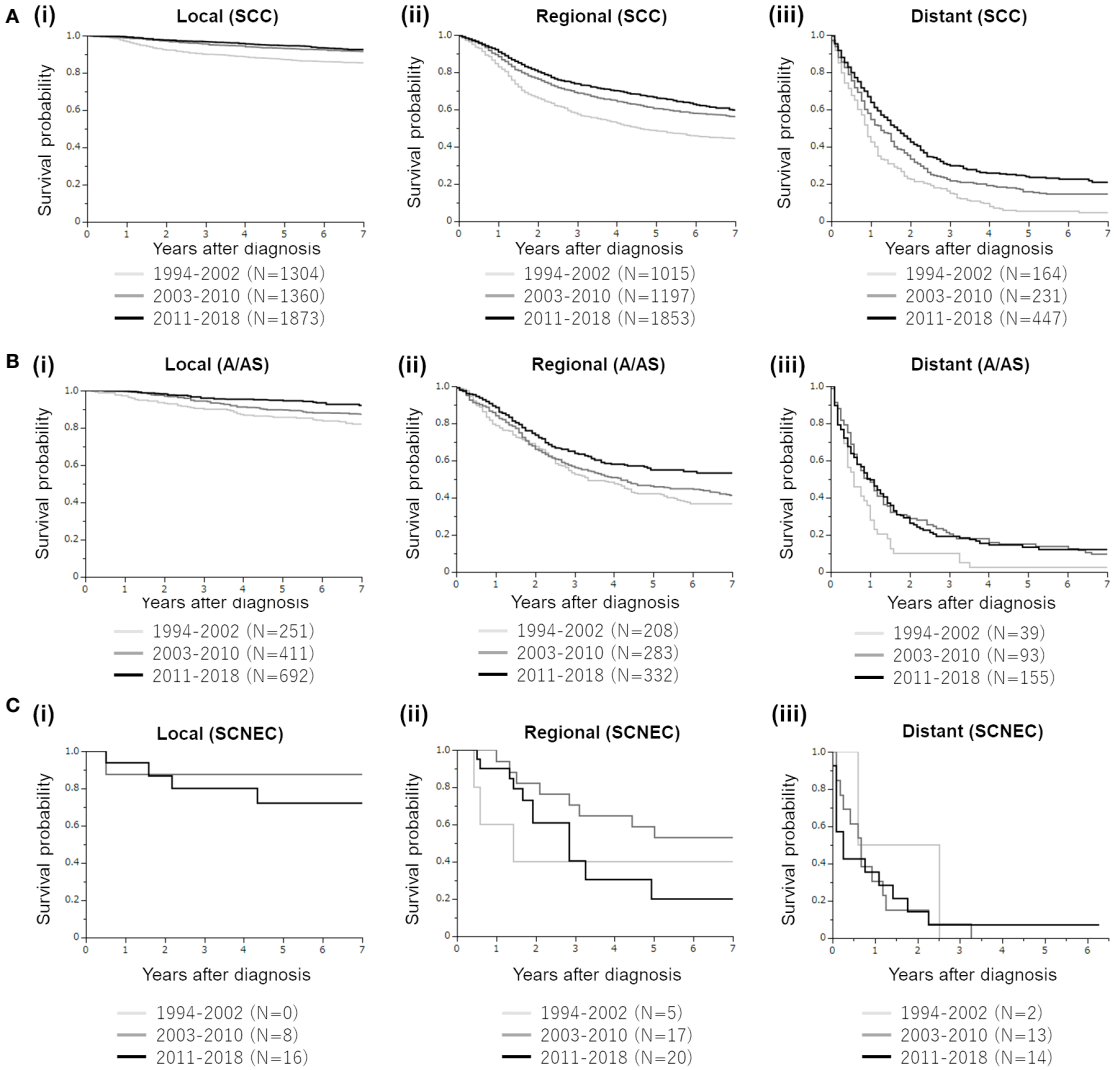
Mortality trends of four major histological subtypes of cervical cancer diagnosed between 1994 and 2018. **(A)** Kaplan–Meier estimates of the overall survival of patients with cervical cancer with squamous cell carcinoma (SCC) histology according to the disease extent and year of diagnosis. (i). Patients with local disease (group 1 [1994–2002] vs. group 2 [2003–2010], p<0.0001; group 1 vs. group 3 [2011–2018], p<0.0001; group 2 vs. group 3, p=0.1948). (ii). Patients with regional disease (group 1 [1994–2002] vs. group 2 [2003–2010], p<0.0001; group 1 vs. group 3 [2011–2018], p<0.0001; group 2 vs. group 3, p=00086). (iii). Patients with distant disease (group 1 [1994–2002] vs. group 2 [2003–2010], p=0.0010; group 1 vs. group 3 [2011–2018], p<0.0001; group 2 vs. group 3, p=0.0090). **(B)** Kaplan–Meier estimates of the overall survival of patients with cervical cancer with adenocarcinoma/adenosquamous cell carcinoma (A/AS) histology according to the disease extent and year of diagnosis. (i). Patients with local disease (group 1 [1994–2002] vs. group 2 [2003–2010], p=0.0557; group 1 vs. group 3 [2011–2018], p<0.0001; group 2 vs. group 3, p=0.0130). (ii). Patients with regional disease (group 1 [1994–2002] vs. group 2 [2003–2010], p=0.3492; group 1 vs. group 3 [2011–2018], p=0.0010; group 2 vs. group 3, p=0.0135). (iii). Patients with distant disease (group 1 [1994–2002] vs. group 2 [2003–2010], p=0.0118; group 1 vs. group 3 [2011–2018], p=0.0082; group 2 vs. group 3, p=0.9047). **(C)** Kaplan–Meier estimates of the overall survival of patients with cervical cancer with small cell neuroendocrine carcinoma (SCNEC) histology according to the disease extent and year of diagnosis. (i). Patients with local disease (group 2 [2003–2010] vs. group 3 [2011–2018], p=0.4711). (ii). Patients with regional disease (group 1 [1994–2002] vs. group 2 [2003–2010], p=0.2921; group 1 vs. group 3 [2011–2018], p=0.9076; group 2 vs. group 3, p=0.0792). (iii). Patients with distant disease (group 1 [1994–2002] vs. group 2 [2003–2010], p=0.5452; group 1 vs. group 3 [2011–2018], p=0.4727; group 2 vs. group 3, p=0.9879).

Regarding SCC histology (**Figure 3A**), patient survival improved with time in patients with regional or distant diseases. Mainly because of the high survival rate, the survival difference between 2011–2018 and 2003–2010 was not statistically significant in patients with local disease (p=0.1948).

Regarding the A/AS histology (**Figure 3B**), improved survival rates according to the year of diagnosis were observed in patients with local disease. In patients with regional A/AC, although the survival curves for 1994–2002 and 2003–2010 were overlapped, the survival of patients in 2011–2018 was significantly improved. In patients with distant (stage IVB) A/AS, although those diagnosed in 2011–2018 or 2003–2010 showed significantly increased survival compared with those diagnosed in 1994–2002, the survival curves of 2003–2010 and 2011–2018 were overlapped, indicating the difficulty in improving the prognosis of patients with distant A/AS.

Finally, regarding SCNEC, in patients with local, regional, and distant diseases, the survival outcome did not improve over 25 years (**Figure 3C**).

## Discussion

In this study, using data from the population-based cancer registry in Osaka Prefecture, we investigated the survival outcomes of patients with cervical cancer with SCNEC, SCC, and A/AS histologies according to the year of diagnosis (1994–2018). We found that the survival outcomes of patients with SCC or local/regional A/AS significantly improved. In contrast, the survival outcomes of SCNEC patients did not improve in these 25 years irrespective of the disease extent. We also found that the survival outcomes of patients with distant A/AS did not improve after 2003. These results highlight the fact that SCNEC (all stages) and distant (stage IVB) A/AS of the uterine cervix are still significant unmet medical needs in Japan.

The precise reasons for these results remain unclear. However, the improved survival outcomes in patients with local and regional SCC and A/AS from 1994 to 2018 (**Figures 3A, B**) can be explained by the following: recent advances in surgical treatment, the development of treatment guidelines that can be widely used in Japan (13), imaging technology to detect small metastases (14, 15), advances in radiotherapy, introduction of cisplatin-based chemoradiotherapy in both adjuvant and definitive settings (16, 17), and extended-field intensity-modulated radiation therapy (18). Although the survival rates of patients with local SCC or A/AS were high (>80%) (**Figures 3A, B**), those of patients with regional SCC or A/AS were not as high (estimated 7-year survival rates of 54.8% and 43.8%, respectively), indicating the need for more effective chemoradiotherapy. A recent phase III trial (OUTBACK trial) demonstrated that the addition of adjuvant chemotherapy to standard concurrent chemoradiotherapy (CCRT) did not improve the survival outcomes of patients with locally advanced cervical cancer (19). Thus, adjuvant chemotherapy after definitive chemoradiotherapy is not recommended for this patient population. Currently, the efficacy of a combination of immunotherapy and chemoradiation is being evaluated in randomized clinical trials (20). Thus, positive results are anticipated.

Improved survival outcomes in patients with distant SCC and A/AS from 1994 to 2018 (**Figures 3A, B**) were presumably due to advances in platinum-based chemotherapy: standard chemotherapy was shifted from single-agent cisplatin (21) to platinum-based doublet (paclitaxel plus either cisplatin or carboplatin) in 2009 (22) and to platinum-based doublet plus bevacizumab in 2014 (23). However, as shown in **Figures 3A, B**, the estimated 7-year survival rates of patients with distant SCC or A/AS remained very low (15.9% and 9.4%, respectively). As demonstrated in a phase III trial (24), we expect that the incorporation of immunotherapies, including an immune checkpoint inhibitor, in the management of distant SCC and A/AS will improve, to some extent, the prognosis of patients today. However, we believe that maximum efforts are required to further improve the survival rate of this patient population.

Because of the rarity of SCNEC histology, there is currently no consensus on effective treatment. In Japan, radical hysterectomy is preferred over definitive radiotherapy for patients with resectable local SCNEC. Postoperatively, adjuvant chemotherapy with etoposide plus cisplatin or irinotecan plus cisplatin instead of radiotherapy is preferred based on a retrospective study conducted in Japan (25). Moreover, a recent Japanese investigation involving patients with IB2 and T1bN1M0 SCNEC suggested that postoperative adjuvant chemotherapy, rather than radiotherapy, improves survival (26). Chemotherapy remains the mainstay of care for patients with advanced SCNEC. Owing to its histopathological similarity to small cell lung cancer (SCLC), chemotherapy for SCNEC of the uterine cervix has been based on SCLC; etoposide plus cisplatin or irinotecan plus cisplatin has been most frequently employed in Japan (27). Despite these efforts, the survival outcomes of patients with in local, regional, and advanced SCNECs did not improve in these 25 years (**Figure 3C**), indicating the limitations of current therapeutic strategies. According to recent reports, SCNEC-specific CCRT using etoposide plus platinum in patients with locally advanced disease (28) or the addition of radiotherapy after chemotherapy in patients with stage IVB disease (29) appears to be effective in patients with SCNEC. Moreover, novel targeting agents based on the distinct genomic profile of cervical SCNECs should be developed.

In the management of preinvasive or invasive cervical diseases, primary prevention through HPV vaccination and secondary prevention through cervical cancer screening are the two most important measures. According to a previous report, the proportion of local and *in situ* stage had increased in Osaka: from 68.5% in 2000–2002 to 81.7% in 2012–2014 for cervical cancer, which might be attributable to the expansion of cancer screening programs (30). The proportion of screen-detected cervical cancer also increased from 14.7% in 2000–2002 to 34.2% in 2012–2014. These are very gratifying. However, the screening rates for cervical cancer in Japan have remained relatively low at approximately 40% (10). Further efforts are undoubtedly required to increase cervical cancer screening rates in Japan. As for HPV vaccination, free vaccination against HPV began in 2010 for Japanese girls and the vaccine was included in the national immunization program since April, 2013. However, in June, 2013, the Japanese Ministry of Health, Labour, and Welfare suspended proactive recommendations for the HPV vaccine after unconfirmed reports of adverse events following vaccination appeared in the media (31). Suspension of proactive recommendations for HPV vaccination had led to a dramatical decrease in HPV vaccination rate and significant increase in HPV infection rates in young Japanese women (32). After a long period of discussion and validation, Japan’s immunization program resumed proactively recommending the use of the HPV vaccine nationwide in April 2022. According to previous reports, greater than 85% of adenocarcinoma and SCNEC were caused by HPV, primarily HPV18 and HPV16 (33). We hope the resumption of HPV vaccination will read to the reduction in the cervical cancer incidence, help solve the unmet needs found in the current study: SCNEC (all stages) and distant (stage IVB) A/AS.

The strength of our study lies in the analysis of long-term cancer registry data, the quality of which has been regarded as meeting international standards (34). During the study period (1994–2018), the proportion of death-certificate-only (%DCO) cervical cancer cases from 1994-2018 was 1.6%. In addition, the proportion of cervical cancer cases microscopically verified (MV%) has been greater than 99% during the study period: 99.1% in 1994-2002, 99.7% in 2003-2010, and 99.5% in 2011-2018. Another strength is that our study is the first to investigate the trends in survival of cervical cancer patients according to tumor histology in association with disease extent in Japan. The limitations of this study should be recognized. First, potential confounders, such as patients’ socioeconomic characteristics, preexisting comorbidities, performance status, postoperative adjuvant treatments, and hospital characteristics, including surgeon volume and infrastructure, were not included in the OCR database. Similarly, the OCR database did not include infection or vaccination statuses of HPV, details of surgery, radiotherapy, or chemotherapy. Second, we could not calculate cancer-specific mortality because of the absence of information on the cause of death in the OCR database. Moreover, as the OCR used the Surveillance Epidemiology and End Results (SEER) Summary Stage for cancer registration and the extent of the tumor was only classified as localized or regional disease, the relative survival rate of SCNEC to SCC or A/AS could not be evaluated according to the FIGO stage or tumor size. Finally, although Osaka Prefecture has a population of approximately 9 million, which is approximately one-thirteenth of the total population of Japan, the present study is not representative of the general population in Japan.

In conclusion, we found that, in contrast to SCC or local or regional A/AS which was associated with improved survival from 1994 to 2018, the prognosis of SCNEC did not improve in these 25 years. Moreover, the prognosis of patients with distant (stage IVB) A/AS did not improve in these 16 years. These results highlight the fact that SCNEC (all stages) and distant (stage IVB) A/AS of the uterine cervix are the significant unmet medical needs in Japan. Therefore, novel treatments should be developed to improve the prognosis of these patients.

## Data availability statement

The data analyzed in this study is subject to the following licenses/restrictions: The use of Osaka Cancer Registry data is restricted only for the approved physicians in Osaka, Japan. It is not publicly available. However, the data that support the findings of this study are available from the corresponding author upon reasonable request. Requests to access these datasets should be directed to Seiji Mabuchi, seiji.mabuchi@oici.jp.

## Ethics statement

The studies involving humans were approved by Research Ethics Committee of Osaka International Cancer Institute. The studies were conducted in accordance with the local legislation and institutional requirements. Written informed consent for participation was not required from the participants or the participants’ legal guardians/next of kin in accordance with the national legislation and institutional requirements.

## Author contributions

Conceptualization, SM. Methodology, SM, TS and NK. Formal analysis, NK. Investigation, TS and NK. Data curation, TM, TS and NK. Writing-original draft preparation, SM and NK. Writing-review & editing, SK. Supervision, IM. All authors contributed to the article and approved the submitted version.
