# Supplementary material for: Twenty-five-year mortality trends of four major histological subtypes of cervical cancer: a population-based study using the Osaka cancer registry data
Source: Front Oncol. 2023 Nov 27;13:1233354. doi: 10.3389/fonc.2023.1233354 (PMC10711201; doi:10.3389/fonc.2023.1233354)
Supplement: Supplementary file 1 [file Table_1.docx]

Supplementary Material

Twenty-five-year mortality trends of four major histological subtypes of cervical cancer: a population-based study using the Osaka Cancer Registry data

Naoko Komura, Seiji Mabuchi, Tomoyuki Sasano, Shoji Kamiura, Toshitaka Morishima, and Isao Miyashiro

*** Correspondence:** Seiji Mabuchi, M.D., Ph.D. [seiji.mabuchi@oici.jp](mailto:seiji.mabuchi@oici.jp)

**Supplementary Materials**

**Supplemental Table 1.** Clinicopathological characteristics of cervical cancer patients according to the histological subtypes.

|  |  | SCC  (Total = 9444)  N (%) | A/AS  (Total = 2464)  N (%) | SCNEC  (Total = 95)  N (%) | p-value |
| --- | --- | --- | --- | --- | --- |
| Age | <39 | 2030 (21.5) | 511 (20.7) | 28 (29.5) | <0.0001 |
|  | 40-60 | 3983 (42.2) | 1246 (50.6) | 48 (50.5) |  |
|  | >61 | 3431 (36.3) | 707 (28.7) | 19 (20.0) |  |
| Extent of disease | Localized | 4537 (48.0) | 1354 (55.0) | 24 (25.3) | <0.0001 |
|  | Regional | 4065 (43.0) | 823 (33.4) | 42 (44.2) |  |
|  | Distant | 842 (8.9) | 287 (11.7) | 29 (30.5) |  |
| Primary Treatment | Surgery | 5380 (57.0) | 1813 (73.6) | 52 (54.7) | <0.0001 |
|  | Radiotherapy | 3045 (32.2) | 333 (13.5) | 24 (25.3) |  |
|  | Chemotherapy | 270 (2.9) | 110 (4.5) | 10 (10.5) |  |
|  | BSC | 660 (7.0) | 190 (7.7) | 9 (9.5) |  |
|  | Unknown | 89 (0.9) | 18 (0.7) | 0 |  |

SCC, squamous cell carcinoma; A, adenocarcinoma; AS, adenosquamous carcinoma; SCNEC, small cell neuroendocrine carcinoma; BSC, best supportive care.

**Supplemental Table 2.** Clinicopathological characteristics of cervical cancer patients with SCC histology.

|  |  | 1994-2002  (Total = 2483)  N (%) | 2003-2010  (Total = 2788)  N (%) | 2011-2018  (Total = 4173)  N (%) | p-value |
| --- | --- | --- | --- | --- | --- |
| Age | <39 | 475 (19.1) | 646 (23.2) | 909 (21.8) | 0.0048 |
|  | 40-60 | 1074 (43.3) | 1177 (42.2) | 1732 (41.5) |  |
|  | >61 | 934 (37.6) | 965 (34.6) | 1532 (36.7) |  |
| Extent of disease | Localized | 1304 (52.5) | 1360 (48.8) | 1873 (44.9) | <0.0001 |
|  | Regional | 1015 (40.9) | 1197 (42.9) | 1853 (44.4) |  |
|  | Distant | 164 (6.6) | 231 (8.3) | 447 (10.7) |  |
| Primary Treatment | Surgery | 1441 (58.0) | 1710 (61.3) | 2229 (23.6) | <0.0001 |
|  | Radiotherapy | 795 (32.0) | 820 (29.4) | 1430 (34.3) |  |
|  | Chemotherapy | 36 (1.5) | 82 (2.9) | 152 (3.6) |  |
|  | BSC | 194 (7.8) | 142 (5.1) | 324 (7.8) |  |
|  | Unknown | 17 (0.7) | 34 (1.2) | 38 (0.9) |  |

SCC, squamous cell carcinoma; A, adenocarcinoma; AS, adenosquamous carcinoma; SCNEC, small cell neuroendocrine carcinoma; BSC, best supportive care.

**Supplemental Table 3.** Clinicopathological characteristics of cervical cancer patients with A/AS histology.

|  |  | 1994-2002  (Total = 498)  N (%) | 2003-2010  (Total = 787)  N (%) | 2011-2018  (Total = 1179)  N (%) | p-value |
| --- | --- | --- | --- | --- | --- |
| Age | <39 | 87 (17.5) | 144 (18.3) | 280 (23.8) | <0.0001 |
|  | 40-60 | 290 (58.2) | 408 (51.8) | 548 (46.5) |  |
|  | >61 | 121 (24.3) | 235 (29.9) | 351 (29.8) |  |
| Extent of disease | Localized | 251 (50.4) | 411 (52.2) | 692 (58.7) | <0.0001 |
|  | Regional | 208 (41.8) | 283 (36.0) | 332 (28.2) |  |
|  | Distant | 39 (7.8) | 93 (11.8) | 155 (13.2) |  |
| Primary Treatment | Surgery | 396 (79.5) | 606 (77.0) | 811 (68.8) | <0.0001 |
|  | Radiotherapy | 69 (13.9) | 109 (13.9) | 155 (13.2) |  |
|  | Chemotherapy | 14 (2.8) | 33 (4.2) | 63 (5.3) |  |
|  | BSC | 19 (3.8) | 35 (4.5) | 136 (11.5) |  |
|  | Unknown | 0 | 4 (0.5) | 14 (1.2) |  |

SCC, squamous cell carcinoma; A, adenocarcinoma; AS, adenosquamous carcinoma; SCNEC, small cell neuroendocrine carcinoma; BSC, best supportive care.

**Supplemental Table 4.** Clinicopathological characteristics of cervical cancer patients with SCNEC histology.

|  |  | 1994-2002  (Total = 7)  N (%) | 2003-2010  (Total = 38)  N (%) | 2011-2018  (Total = 50)  N (%) | p-value |
| --- | --- | --- | --- | --- | --- |
| Age | <39 | 3 (42.9) | 12 (31.6) | 13 (26.0) | 0.7165 |
|  | 40-60 | 2 (28.6) | 18 (47.4) | 28 (56.0) |  |
|  | >61 | 2 (28.6) | 8 (21.1) | 9 (18.0) |  |
| Extent of disease | Localized | 0 | 8 (21.1) | 16 (32.0) | 0.3318 |
|  | Regional | 5 (71.4) | 17 (44.7) | 20 (40.0) |  |
|  | Distant | 2 (28.6) | 13 (34.2) | 14 (28.0) |  |
| Primary Treatment | Surgery | 3 (42.9) | 19 (50.0) | 30 (60.0) | 0.6839 |
|  | Radiotherapy | 3 (42.9) | 11 (29.0) | 10 (20.0) |  |
|  | Chemotherapy | 1 (14.3) | 5 (13.2) | 4 (8.0) |  |
|  | BSC | 0 | 3 (7.9) | 6 (12.0) |  |
|  | Unknown | 0 | 0 | 0 |  |
